# Specialty grand challenge for molecular and structural endocrinology: celebrating successes and the future ahead

**DOI:** 10.3389/fendo.2025.1756684

**Published:** 2026-01-02

**Authors:** Debbie C. Thurmond

**Affiliations:** Department of Molecular & Cellular Endocrinology, Arthur Riggs Diabetes and Metabolism Research Institute of City of Hope, Duarte, CA, United States

**Keywords:** allosteric interactions, G protein-coupled receptors, hormone receptors, ligand, signaling, structure-function

## Introduction

Discovery of what is now referred to as “the canonical insulin receptor signaling cascade,’ and other hormone receptor signaling cascades, was made possible by the pivotal findings of giants in the field in structural biology, molecular biology, biochemistry, and cell biology. Fundamental techniques in these fields led to the isolation and purification of these hormone receptors and their ligands, to generate these proteins via bacterial and insect cell expression, to identify and test mutations in the extracellular ligand binding domains and intracellular activation domains, and to discern key intracellular signaling pathways that brought about breakthroughs in drug design to benefit patients with endocrinopathies. Recent years have seen molecular and structural endocrinology leaps forward in the battle against obesity and obesity-related diseases, through seminal research on G protein coupled receptors and their ligands, specifically the incretin class of receptors such as GLP-1 receptor (GLP-1R), GIPR and GCGR. These receptor agonists are best known as GLP1-RAs. Molecular ‘tinkering’ using state-of-the-art technologies now deliver greater potency of GLP1-RAs to patients. These events of the prior 50 years are certainly worthy of celebrating molecular and structural endocrinology.

So, are we done now? Absolutely not. The challenges with GLP1-RAs are to make them as dual and triple receptor agonists. In addition, subcellular compartmentalization of the incretin receptor signaling is becoming increasingly important, as it impacts efficacies of the various GLP-1RAs. There are additional emerging understudied G protein coupled receptors, which are clearly demonstrated to be worthwhile drug targets of engineered GLP-1RAs. Improved engineered forms of insulin remains vibrant, serving the needs of patients living with autoimmune diabetes (type 1 diabetes) as well as patients with insulin-dependent type 2 diabetes. Beyond these examples, underexplored receptors and non-receptor proteins that respond to endocrine hormones, and their reactions to metabolic changes that evoke endocrinopathies, are ripe for fundamental investigations of potential allosteric interactions and drug targeting thereof. In our new world of data science capabilities, AI and computational therapeutics, molecular and structural endocrinology will catapult new drug targets that have not been ‘accessible’ in the past (1). Clinical progression in tackling endocrinopathies via foundational drug discovery and development made possible through molecular and structural endocrinology breakthroughs is our call to action for the future ahead.

## A rich history, and our call to action in the field of molecular and structural endocrinology

With the widespread use of GLP1-RAs, are obesity and related diseases (diabetes, aging, cardiovascular disease, Alzheimer’s disease, etc.) soon to be ‘cured’? This is a complex question indeed. While the worldwide prevalence of obesity has more than tripled between 1975 and 2022, with obesity incidence at 1 billion, and overweight + obesity totaling nearly 3 billion worldwide (2), these drugs are certainly a leap forward in potential to curb obesity-linked diseases. Yet, there remains an unmet need for alternative and adjuvant therapies, particularly for the aged, and for those patients with ‘lean’ type 2 diabetes. Indeed, the patient populations of the aged, and of those with non-obesity-linked type 2 diabetes, are the fastest growing populations in the type 2 diabetes field today, globally (3).

The success of the GLP1-RAs has spectacularly demonstrated to us that G protein coupled receptors have tremendous value as potential drug targets, and many that have been implicated in endocrinopathies that remain understudied and underdeveloped. Small molecules, engineered ligands, molecular interrogation of structure and function and contributions to existing or perhaps new intracellular signaling cascades, are opportunities for drug development efforts in endocrinology. Computational therapeutics, integrating machine learning and AI in drug discovery and development, are already in use as newly sharpened tools for speed, precision, and maximizing these opportunities.

Lesser developed opportunities for intervention exist in pursuing allosteric interactions, particularly in non-liganded cell-surface receptors, and key protein-protein interactions, binary or multimeric, that are the gatekeepers of key events in endocrinology, such as hormone release from specialized endocrine cells, or responsiveness to metabolic stimuli, including unhealthy stimuli. Advancements in molecular and structural biology technologies, high-throughput screening modalities, and rapid nucleic acid and peptide/protein engineering capabilities, coupled with computational therapeutics, opens new doors in our field to capitalize on these fundamentally important allosteric interactions beyond the academic realm, into the realm of possibilities for clinical application to resolve endocrinopathies in the future.

Testing modalities, *in vitro*, ex vivo and *in vivo*, are changing today and the importance of human-derived organoids has emerged and poised to position, or perhaps out-position, that of the *in vivo* animal models of the past and present. With organoids rises the capability for overcoming challenges with species-specific responsiveness (e.g. working in rodents and primates but failing to work in humans). In addition, organoids afford the ability to molecularly tinker, via CRISPR, for cellular therapeutic advancements. This also raises the possibility for personalized medicine approach, using the patient’s own cells. Notably however, sole reliance upon organoids for drug testing will lack the discernment of multiorgan effects that are discovered by incorporating *in vivo* model testing modalities, and caution must be taken on the interpretation of organoid-only drug testing.

The field of molecular and structural endocrinology is founded on the shoulders of giants, and this has and should continue to be celebrated, studied, and revered. These ‘giants’ used out-of-the-box thinking, thought broadly and worked with focus to move our field forward, which led to monumental advancements (aka, the creation of the biotech industry (4),). One might surmise that the expectation, and hope, of these giants is that we continue this push forward and upward, embracing opportunities in our field, integrating technological advancements made in other fields as well as our own, and embracing team science for maximal creativity and discovery success.
